# State-of-the-Art Water Treatment in Czech Power Sector: Industry-Proven Case Studies Showing Economic and Technical Benefits of Membrane and Other Novel Technologies for Each Particular Water Cycle

**DOI:** 10.3390/membranes11020098

**Published:** 2021-01-30

**Authors:** Jaromír Marek

**Affiliations:** Department of Chemistry, Faculty of Science, Humanities and Education, Technical University of Liberec, Studentská 1402/2, 461 17 Liberec, Czech Republic; jaromir.marek@mail.com; Tel.: +420-732-277-183

**Keywords:** water treatment, power generation, heating station, energy, boilers, ion exchange, membrane processes, reverse osmosis, ultrafiltration, electrodialysis, electrodeionization, shock electrodialysis, membrane distillation, capacitive deionization, forward osmosis, OpEx, CapEx, payback period, turbine condensate, SDI, colloidal particles

## Abstract

The article first summarizes case studies on the three basic types of treated water used in power plants and heating stations. Its main focus is Czechia as the representative of Eastern European countries. Water as the working medium in the power industry presents the three most common cycles—the first is make-up water for boilers, the second is cooling water and the third is represented by a specific type of water (e.g., liquid waste mixtures, primary and secondary circuits in nuclear power plants, turbine condensate, etc.). The water treatment technologies can be summarized into four main groups—(1) filtration (coagulation) and dosing chemicals, (2) ion exchange technology, (3) membrane processes and (4) a combination of the last two. The article shows the ideal industry-proven technology for each water cycle. Case studies revealed the economic, technical and environmental advantages/disadvantages of each technology. The percentage of technologies operated in energetics in Eastern Europe is briefly described. Although the work is conceived as an overview of water treatment in real operation, its novelty lies in a technological model of the treatment of turbine condensate, recycling of the cooling tower blowdown plus other liquid waste mixtures, and the rejection of colloidal substances from the secondary circuit in nuclear power plants. This is followed by an evaluation of the potential novel technologies and novel materials.

## 1. Introduction

### 1.1. The Current State of Power Industry Water Treatment in Eastern Europe

**Figure 1:**
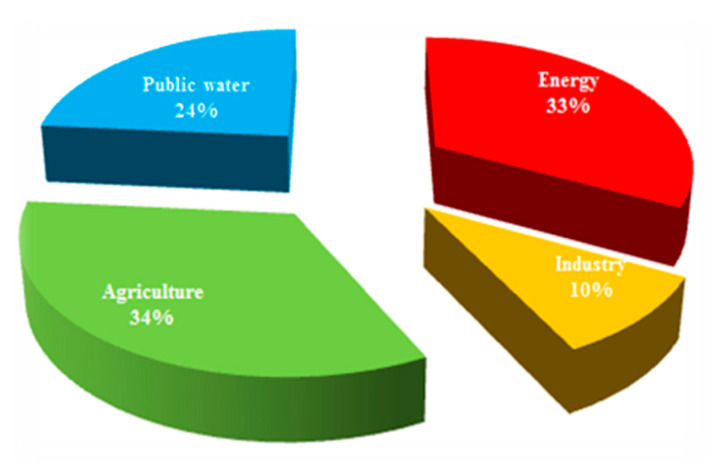
Water consumption in the whole of Europe, based on Eurostat data (2020). Turkey is included [2].

According to the European Environment Agency (2020), power generation represents the second largest overall share of raw water consumption in Europe (Figure 1) in all sectors (including public consumption, agriculture and industry). The greater overall share of agriculture is due to the more agricultural nature of Southern Europe, but in Eastern Europe power generation plays the major part. Therefore, it is desirable to start in the wide field of the power and heat generation (i.e., energetic) sector in order to maximize the effectiveness of water recycling, minimize the amount of waste-water, and make its treatment ecological and economical.

The main reason for water treatment in power generation is to avoid the corrosion and scaling of the whole system (including boiler, piping and turbine). The total volume of water cycles (including boiler circuit and cooling circuit) in one power or heating station usually ranges from thousands to tens of thousands of cubic meters per hour. A very rough approximation of the total volume of water in power stations (presented in this study) can be assumed as follows: 1 kg of condensated steam requires approximately 50–60 kg of cooling water (m_cw_)—depending on the change in steam enthalpy (h) behind the turbine [1]:

(1) $$m_{\mathrm{cw}}=\frac{\Delta h}{\mathrm{cp}\Delta t}$$

∆t is generally 10 °C, the specific heat capacity of water (c_p_) is 4.19 kJ kg^−1^, and the rough value of the enthalpy of steam in front of the turbine is 2900 kJ kg^−1^ and behind the turbine 750 kJ kg^−1^, so the change in enthalpy ∆h is roughly 2150 kJ kg^−1^.

The water needs of power and heating stations in Czechia range from 0.372 m^3^ MWh^−1^ (Heating Station Písek) to 2.622 m^3^ MWh^−1^ in Třinec Heating Station [2,3].

The following diagram (Figure 1) shows the statistical data of water consumption per sector in the whole of Europe, and Table 1 shows the detailed data for each part of Europe. The data have been provided by the European Environment Agency [2].

Ion exchange technology still takes the major share in water treatment in the currently operating power stations and heating plants in Eastern Europe. However, the first exceptions have appeared, namely in the newly built power plants (in Hungary, Poland, Romania or the newly built Turkish Yunus Emre), and in Czech central heating plants, where the economy and environmental protection were of the greatest interest, and therefore membrane technologies have been employed in water treatment. Membrane technologies are even more common in small plants (with low-pressure boilers or small cooling circuits) where technological steam is produced (e.g., bakeries, breweries). However, these small plants use the dosing of antiscaling and anticorrosion chemicals in the majority of cases. Furthermore, mid- and high-pressure boilers in power and heating stations use alkalization of boiler water (because demineralized water is so-called “hungry water”), to shift the value from the corrosion area of Langelier Saturation Index (LSI) to passivated region. Cooling circuits in power plants with cooling towers employ dosing of antiscaling and anticorrosion chemicals. However, this is not I the focus of this study.

The work is primarily divided into the three main areas of water treatment in power industry—the treatment of make-up water for boilers, the recycling of cooling tower blowdown, and the treatment of specific types of water (the recycling of turbine condensate, the recycling of liquid waste mixtures, the recycling of boric acid in the primary circuit in nuclear power plants, etc.).

**Figure 2:**
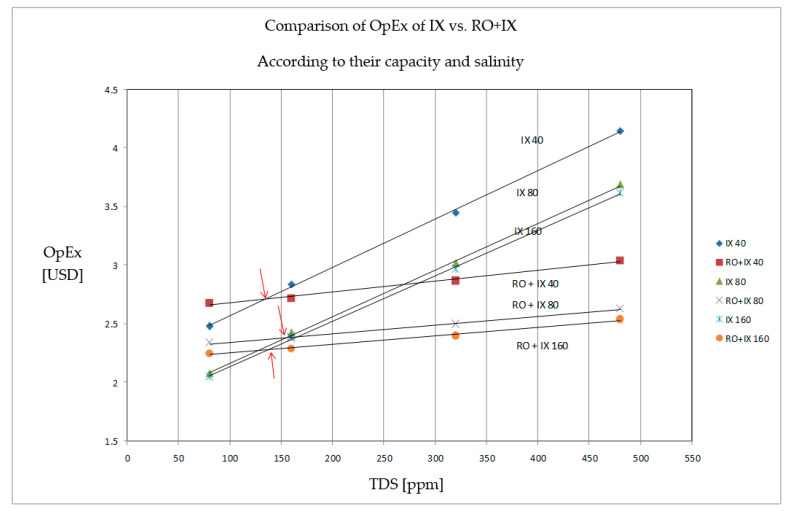
Comparison of OpEx of IX (ion exchange) technology and IX + RO (reverse osmosis) by Beardsley, DOW company (1994), according to TDS and capacity (40, 80 and 160 m^3^ h^−1^) [4].

The conception of membrane processes, which compete with the whole ion exchange technology for water treatment in power and heating stations, was first described and economically compared by Beardsley (Dow company) in 1995 [4], and later by Dardel (2005) [5], as seen in Figure 2.

In the last 10–15 years there has been a huge drop in the investment costs of membrane processes due to their widespread use. Unfortunately, this does not mean that the key industries using water treatment technologies have switched to membrane technology [6,7,8]. 

The combination of reverse osmosis (RO) followed by ion exchange columns as seen in Figure 2 is not optimal anymore, simple explanation is in Section 2.3 (contamination of RO permeate by ppb of colloidal particles from ion exchange resin). However it is still in the operation (e.g., in Supercritical unit of Power Station Ledvice, Czechia, from 2012) and still being designed for new systems [6].

### 1.2. The Choice of Membrane Technologies for Water Treatment in the Power Industry

The current membrane technology market provides a wide portfolio of products. The power industry demands highly reliable technologies—e.g., pressure membrane processes (ultrafiltration, reverse osmosis, nanofiltration, microfiltration) and electromembrane processes (electrodialysis and electrodeionization), which are known and have been proven by tens of years of operation [7,8,9]. The technologies newly developed in recent years (e.g., membrane distillation, capacitive deionization, forward osmosis, etc.) are still not reliable enough to ensure problem-free, economically effective, and environmentally friendly long-term operation (according to the statements of power stations managers) [7,10].

When choosing whether to use ion exchange technologies, membrane processes or their combination, the major aspect is economics. The capital expenditure of membrane technology has already become comparable to the ion exchange technology. Membrane technology can save from hundreds of thousands to millions of Czech crowns (tens to hundreds of thousands EUR) in operating expenses in a standard heating plant (up to 100 m^3^ h^−1^) annually. This option is convenient for all the following cases: newly built power plants, existing plants (where the resin is at the end of its operating lifetime), and plants which can reduce the cost of additional water treatment by integrating membrane separation technology [8].

The main argument for the selection of ion exchange technology by technological engineers in power and heating plants is its reliability, and the time for which the technology has been in use (nearly 100 years), so the aim of this study was to demonstrate the uptime and reliability of membrane technology in pilot tests in power plants and heating plants, and make an economic comparison with ion exchange technologies.

### 1.3. The Novelty of the Contribution

Despite the fact that the article is conceived as an overview of water treatment technologies in real operation, its novelty lies in the few following technological models optimized within this work: (1) the treatment of turbine condensate, (2) the recycling of cooling tower blowdown, (3) the recycling of other liquid waste mixtures, and (4) the rejection of colloidal substances from secondary circuit in nuclear plants. 

Last is included a brief evaluation of potential novel methods and materials.

However, the most important target of this work is complex summarization of common and novel methods of water treatment in power sector.

## 2. Water Cycles and Their Type of Water Treatment

### 2.1. Make-Up Water for Boilers

In the majority of cases, surface water—mainly from rivers or lakes—serves as the source of make-up water for boilers. This is why the ion exchange technology or membrane technology, both used for desalination, also require pretreatment. The pretreatment consists of rough filtration (racks and seeves), soft filtration (sand and activated carbon filters) and coagulation with flocculation. As the experiment has shown, these steps are unavoidable for both the ion exchange and membrane processes [1,6,11].

#### 2.1.1. The Standard Ion Exchange Technology

**Figure 3:**
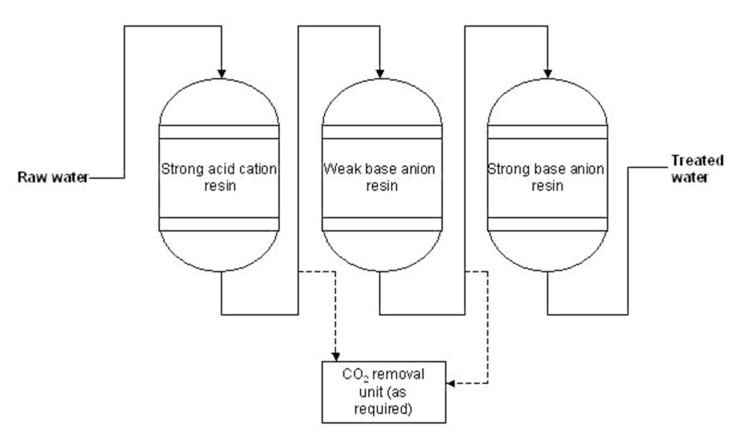
Demineralization technology—Ion exchange units by Lenntech company [9,13].

The make-up water for boilers, when prepared by the ion exchange technologies, mostly uses demineralization lines. These are four, three or two-stage column lines (depending on inlet water quality). Four-stage lines are composed of a strong acid cation exchanger followed by a weak acid cation exchanger, followed by a weak base anion exchanger and a strong base anion exchanger. In case of three-stage demineralization (Figure 3), the strong acid cation exchanger is followed only by a weak and a strong base anion exchanger, and in two-stage demineralization the strong acidic cation exchanger is followed just by the strong base anion exchanger. 

Figure 3 describes ion exchange technological scheme in Heating station Liberec, Czechia (see case study in Section 2.1.3.2). Consumption of chemicals for this station (based on river Nisa) is summarized in Table 2a,b. Half year is ideal predicative time period, including winter months (when the surface water in Czechia is contaminated by NaCl and CaCl_2_, technical grade, used for melting snow on the roads) and summer months (when the rivers and other surface sources are dried, i.e., concentrated by summer heat) [6,8,11].

In the preparation of make-up water in deionization lines, two-stage lines are made of strong acid cation exchanger followed by weak alkali anion exchanger. The conductivity of water and the content of SiO_2_ are the most important monitored parameters to which attention must be paid, according to the guidelines for make-up water for boilers (VGB-R 450 Le—The European Technical Committee, 2011 [12]).

#### 2.1.2. The Membrane Technology

The substitution of demineralization or deionization (ion exchange) technology for the preparation of make-up water for middle- and high-pressure boilers (≥8 MPa) always requires the combination of reverse osmosis and electrodeionization. Reverse osmosis (RO) can be applied in the desalination of water only in low-pressure boilers (≤8 MPa, operating temperature up to 115 °C, with output over 60 kW, due to Czech Standard—ČSN 07 7401) where the conductivity of make-up water is not limited. However, for middle- and high-pressure boilers, the conductivity is limited to 0.2 μS cm^−1^ by Czech Standard ČSN 07 7403, and is recommended to be 0.1 μS/cm by European Standard VGB-R 450-Le [12] and by EPRI (Electric Power Research Institute)-Guidelines for Preservation, Layup, and Startup of Water Treatment Equipmen (ID 3002007941) and Guidelines for makeup water treatment (ID 1019635) [14,15]. That means it is essential to use at least single pass RO together with electrodeionization (EDI). In the ideal case, double-pass RO (where the permeate from first pass becomes feed to second pass, to ensure the maximum elimination of hardness and colloidal particles) followed by EDI is used. When installing reverse osmosis, it is essential to make sure the inlet stream SDI_15_ ≤ 5 (silt density index measured using a cellulose filter with 450 nm pores, 1 bar pressure and 15 min period). If the inlet water SDI_15_ > 5, pre-installation of an ultrafiltration unit is required. 

**Figure 4:**
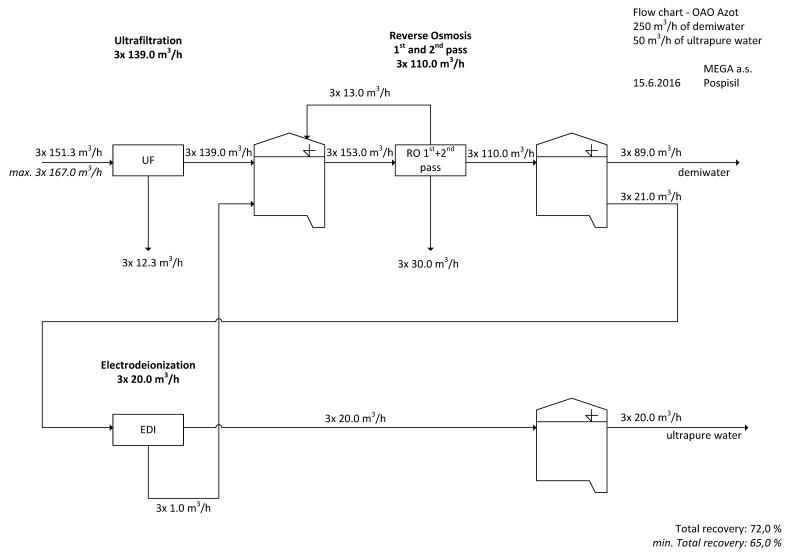
Example of industrial design of preparation of ultrapure make-up water for boilers using membrane technology, Mega Group (Czechia) [6,15,16,17]. The scheme shows water needs of membrane processes which is reflected to higher water cost compared to Ion Exchange technology. That is also the reason to recycle waste streams as much as possible as seen in this picture.

In the majority of cases, these are ultrafiltration units (UF) with hollow fibers, with backflush set to 30–45 min. The basic technology scheme and water capacity and water conversion are illustrated in Figure 4.

In the beginning of replacing the standard technologies (filtration, coagulation and ion exchange), there were efforts to replace coagulation and flocculation by ultrafiltration or microfiltration units. However, very soon after the first installations, the experience showed that neither microfiltration nor ultrafiltration can replace standard coagulation and flocculation. The ultrafiltration units got clogged very easily, and their performance decreased rapidly after installation when the coagulation and flocculation (i.e., clarification) were avoided. Finally, it was proven that coagulation and flocculation are very effective and low-cost operations compared to ultrafiltration/microfiltration, with very high crossflow and frequent backflushes. Regardless, ultrafiltration (more frequently than microfiltration) is a very desirable process for the protection of the downstream reverse osmosis membranes in order to increase reverse osmotic effectivity and prolong the lifetime of the membranes [6,9,10].

#### 2.1.3. A Comparative Pilot Test of Membranes and Ion Exchangers on Make-Up Boiler Water

The most important case study was carried out on the make-up water for boilers. This water represents the highest standards of quality and strict limits of conductivity, concentration of ions, suspended and colloidal solids and dissolved gasses, as determined by national or European standards (in the European Union) [12]. The conductivity of make-up water for high-pressure boilers should be as close as possible to theoretical water (i.e., clear water containing just H_2_O) exhibiting a resistivity of more than 17 MΩ cm^−1^. The arrangement of membrane units in the pilot test was inspired by the combination of membrane processes commonly used in western European countries. These consist of ultrafiltration, reverse osmosis and electrodeionization. The main purpose of an ultrafiltration unit is to decrease the silt density index (SDI_15_ < 5) before feeding the reverse osmosis. The reverse osmosis substitutes classical ion exchange demineralization units. The downstream electrodeionization unit then completes the desalination process and polishes the water.

The technology of electrodeionization substitutes mixed beds, because it avoids the additional problems with the chemicals used in the regeneration of mixed beds (e.g., liquid wastes, neutralization of wastes), administration, the transportation of chemicals and wastes, large built-up area, semi-automatic operation, etc. Electrodeionization (EDI) is intended for polishing the reverse osmosis (RO) permeate. It is an essential process which cannot be simply substituted by two-pass (or more) reverse osmosis. The two-pass reverse osmosis usually serves for sequential decarbonization and desilication. The conductivity of the osmosis permeate from the second pass is approximately 1 μS cm^−1^. The energy consumption of two-pass reverse osmosis is higher in comparison with electrodeionization, and the recovery rate is also lower. Two-pass reverse osmosis is used when the hardness of raw water is too high, or when we want to achieve a water quality after electrodeionization that is better than 16 MΩ cm^−1^ (<0.0625 μS cm^−1^). Therefore, the electrodeionization process is unavoidable in the preparation of the make-up water for middle- and high-pressure boilers.

##### 2.1.3.1. Small-Capacity Heating Station Michle (35.5 MW, 6 MWe)

**Figure 5:**
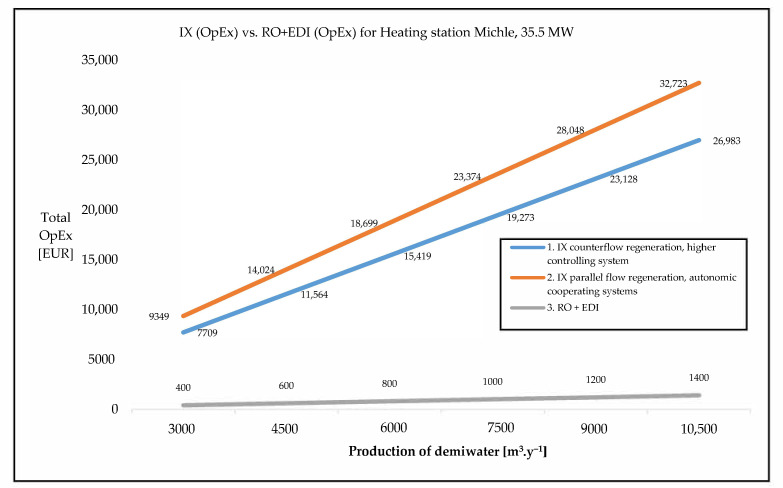
OpEx of IX vs. RO+EDI for small heating station Michle, Pražská teplárenská Ltd.

The first case study was only a theoretical study, represented by small-capacity heating station Michle, Pražská teplárenská company, calculated by the company Memsep [10]. The data in the tables below (Table 3 and Table 4 and Figure 5) were presented in an opened tender. The winner of this selection procedure was the SES Bohemia Engineering company with Septron RO + EDI technology. The data of the winner are not published for obvious reasons. The other competitors were Mega and Culligan. The prices of the technology submitted by the Mega company included complete equipment (tanks, pumps for transportation of water from the source, piping, dosing of chemicals, cartridge filters, etc.), so the price was 1.6× higher compared to Culligan. However if we subtract this periphery, the CapEx of the membrane technology is quite similar for both companies.

This case study presents A rough approximation of A comparative study of membrane technology and ion exchange technology. The data in the study were projected by an authorized appraiser [10]. Real data measured and collected in industrial operation are in the following paragraph. 

##### 2.1.3.2. Heating Station Liberec (182 MW, 7 MWe)

**Figure 6:**
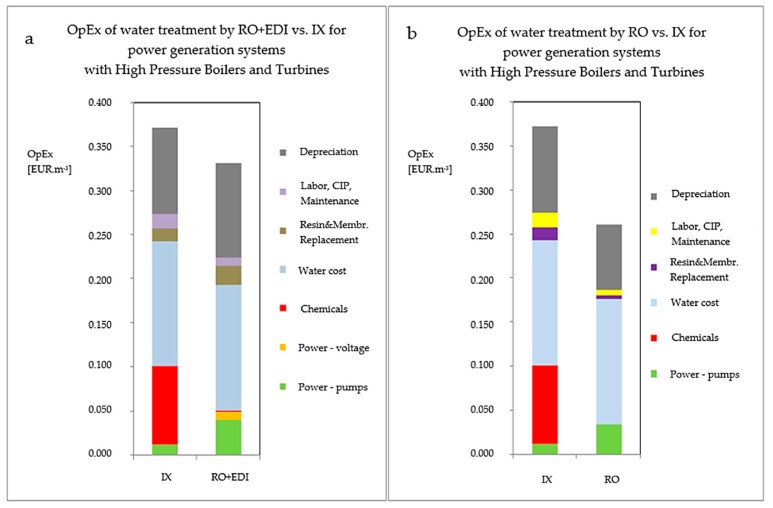
The total cost of 1 m^3^ of water treated by membrane technology compared to ion exchange technology for 90 m^3^ h^−1^ in a system with (**a**) and without (**b**) a turbine. Heating station Liberec. The water source is river Nisa. Both cases requires sufficient pretreatment to avoid utilization of UF (racks and seeves, followed by high quality spiractor or flotation for efficient clarification, and finally two stage sand filters–first with rough and second with soft sand).

This study was carried out on the make-up water for boilers by the MemBrain company in Liberec Heating Station. The pilot membrane units’ capacities were 2 and 8 m^3^ h^−1^. The capacity of ion exchange technology (IX) was 90 m^3^ h^−1^.The results for the OpEx comparative study of IX vs. membrane technology are depicted in the following Figure 6 and Table 5. The water source is river Nisa. Both cases requires sufficient pretreatment to avoid utilization of UF (firstly racks and seeves, followed by high quality spiractor or flotation for efficient coagulation and clarification, and finally two stage sand filters–first with rough sand i.e., rapid sand filters and second with soft sand i.e., slow sand filters).

**Figure 7:**
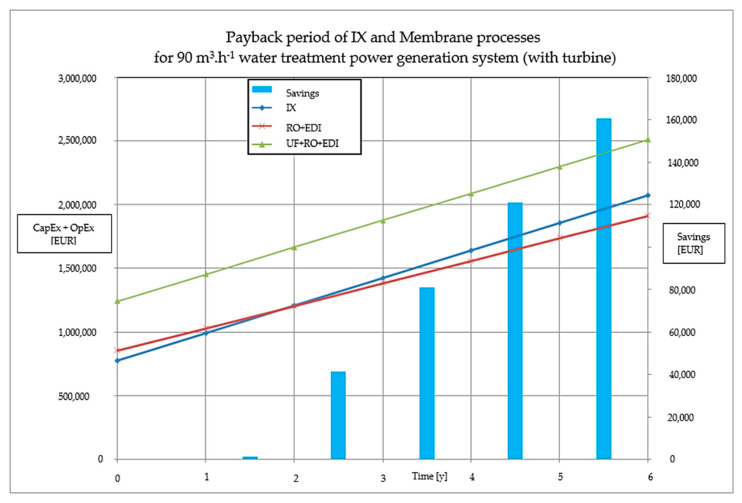
The payback period of water treatment in a power plant with 90 m^3^ h^−1^ of make-up water. Comparison of ion exchange and membrane processes.

Although the difference between the column representing the RO + EDI and the one standing for ion exchange in Figure 6a is not significant, it constitutes accumulated operating expense savings worth tens to hundreds of thousands of Euros annually. The accumulated savings are represented by the blue column in Figure 7.

The ultrafilatration unit was originally meant to substitute the process of coagulation and flocculation (i.e., clarification). Despite its purpose, the actual industrial experience revealed the indispensability of the mentioned processes [18]. As such, nowadays, ultrafiltation is used to protect reverse osmosis membranes and to prolong the lifetime of osmosis membrane modules. However, the pilot test evaluation showed the economical pointlessness of the inserted ultrafiltration process. If the ultrafiltration was cut out and the lifetime of osmosis modules was as much as halved (2 years), then both the capital expenditure and the operating expenses still would be lower (Figure 1) in comparison to membrane technology including ultrafiltration. However, there exist cases wherein the use of ultrafiltration is inevitable, even after optimal coagulation (SDI_15_ > 5).

Figure 7 shows the well-known fact that even if the capital expenditure of membrane technology is higher in comparison to ion exchange technology, the investment returns through the lower operating expenses in 2 or 3 years. The savings in the operating expenses for the 90 m^3^ h^−1^ system are well over EUR 50,000 annually. Membrane technology provides many advantages compared to ion exchange columns. 

According to Figure 2, there are some limits for the utilization of membrane technologies. The area of application is determined by the salinity of raw water (i.e., the lower limit). The value of salinity in the presented case study was around 150 ppm of dissolved salts (i.e., very low concentration). The concentration of salts in common surface water is higher in the majority of cases. Considering that surface water is used in power generation, it can be assumed that the membrane processes are always more suitable than ion exchangers (when they was suitable in the presented case with such a low concentration).

The electromembrane processes are irreplaceable in water treatment in power generation. We need to apply electrodeionization for the make-up water for boilers (to meet the water quality requirements recommended by Ref. [12]). 

### 2.2. Turbine Condensate Treatment–Heating station Chomutov (84 MW, 20 MWe)

Turbine condensate is condensed boiler steam, so theoretically it should be the same quality. However, in real-life operation, the water erodes the surface layer of piping, so it contains iron (in many forms, even suspended particles), as well as traces of ions from the alcalization of boiler water and air gases—that is why the conductivity of condensate is slightly higher (approximately 1.5 μS cm^−1^) compared to boiler make-up water (<0.2 μS cm^−1^ for heating station). The condensate is usually recycled through sand filters and a strong acid cation exchanger, which captures solid particles and cations of iron. For this purpose, we tested EDI (replacement of a cation exchanger column) with a pretreatment with replaceable standard cartridge filters. 

**Figure 8:**
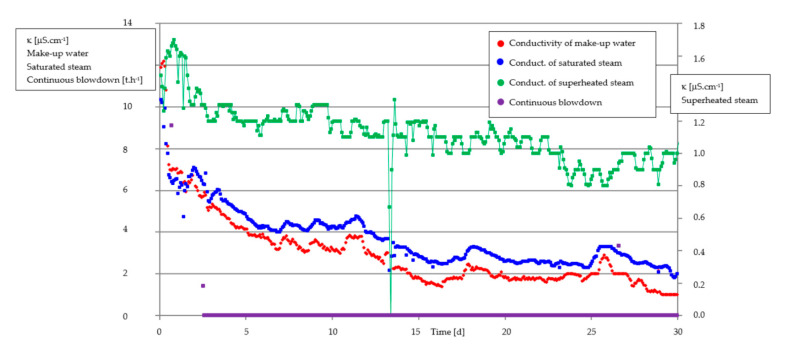
The recycling of turbine condensate by EDI with continual blowdown (violet line, covering x axis) closed 3 days after applying EDI.

The result of this pilot test was quite outstanding. In the Chomutov heating station (Actherm Chomutov), such a good quality of recycled condensate was achieved that it enabled closing the continuous blowdown completely (Figure 8), and even after the closing of the continuous blowdown, the quality of boiler water (and treated condensate and other streams) still kept improving (in the range of days).

The calculation of operational expenses shows additional savings due to the heat loss of continuous blowdown in standard operation (without EDI). It amounts to EUR 3668 annually in heat, and EUR 6058 annually in water savings. Obviously, it is not an astronomical amount, but in fact it is only one of many other additional benefits of membrane treatment in the power industry.

### 2.3. Decreasing TOC in Piping System–Temelín Nuclear Power Plant (3000 MW, 2168 MWe)

**Figure 9:**
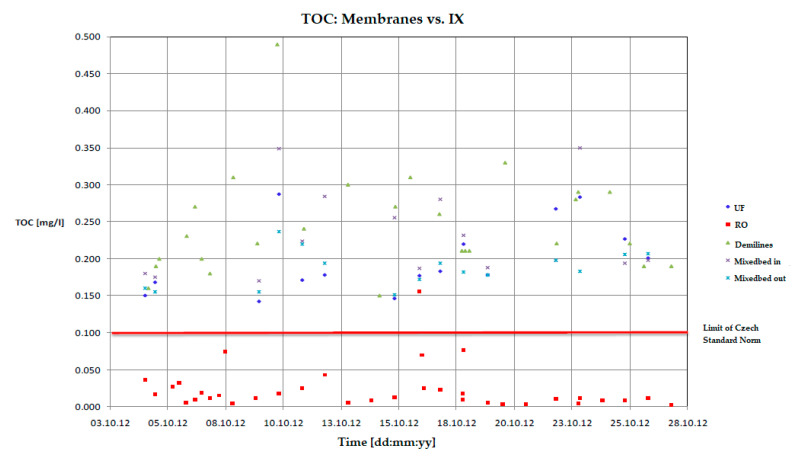
Decreasing TOC in demineralized water outlet from mixed bed columns in Temelín Nuclear Powerplant (CEZ group), secondary circuit. TOC online analyzer Mettler Toledo 6000TOCi.

Another pilot test of membrane technology took place in the Czech Temelín Nuclear Power Plant (ČEZ group). The aim was to avoid the clogging of the valves in the secondary circuit. It was presumed that the clogging was caused by the high content of TOC (total organic carbon), which the current ion exchange technology failed to capture. The online water analysis of TOC confirmed the higher concentration of TOC than is recommended by the Czech ČSN EN 60964 Standard (derived from European IEC 60964 Standard), namely 0.1 mg L^−1^. The outlet from demineralization ion exchange lines (“demilines,” see Figure 3) was measured first, followed by the outlet from mixed bed lines. Obviously (as seen in Figure 9), both lines cross the limit of the Czech Standard—the concentration ranged from 0.120 to 0.260 mg L^−1^ TOC. The proposed solution was to have the demiline product treated by the ultrafiltration unit. The pilot UF unit had a module of hollow triacetate fibers (Microdyn-Nadir Aquadyn FT-50-AC) with the pore size of less than 15 nm. The results of this case study are visible in the following picture (Figure 9). Obviously, the UF effectivity was negligible, and the limits were not met again. 

For the next pilot test, RO with Hydranautics ESPA-2-LD-4040 modules from the Nitto Denko company (modules highly resistant to fouling by organic colloidal substances) was used. As seen in Figure 9, the achieved average concentration of TOC was well below the limit of the Czech Standard, and it was 0.022 mg L^−1^ for the treated demiline product. The RO was also tested as a method for the clarified water (i.e., after coagulation and flocculation) in combination with UF pretreatment (to keep SDI_15_ < 5), and the results were also outstanding—0.033 mg L^−1^. 

It was also observed that when the RO permeate was sent to mixed beds columns, the TOC behind the mixed bed exceeded the limit again. This means that the ion exchange lines leak low molecular organic compounds and pollute the permeate. This is a very important outcome of the study, revealing that the combination of RO + EDI is more appropriate for the future production of high-quality ultrapure water, contrary to the combination of RO + mixedbed.

### 2.4. Recycling Cooling Tower Blowdown

There are many countries all around the world (especially in the Near East) suffering from the lack of water resources, who are thus being pushed to save as much water as possible. They have to recycle cooling tower blowdown and waste-water. The second case of waste-water and cooling water blowdown treatment occurs in small plants, which use water from the municipal water distribution network. In this case, the reason is the price of the water, as recycling can save a significant amount of money. The third example is the expansion of a power plant or a heat station, rendering the current source of water insufficient and resulting in the lack of make-up water.

Electrodialysis is the most suitable process for the treatment of this type of water. First, the requirements concerning feed water pretreatment are less strict for electrodialysis in comparison with reverse osmosis (a sand filter is usually sufficient). The salinity of the product (diluate) from electrodialysis is commonly comparable with the salinity of drinking water (between 0.1 and 1 mS cm^−1^). The LSI thus does not reach the corrosion values, which is the requirement for cooling circuits. This case of cooling water blowdown treatment was validated on the technology with the capacity of 210 m^3^ h^−1^, realized by Mega company in Arak, Iran (Gemwater) and capacity of 425 m^3^ h^−1^ realized again by Mega in Rio De Janeiro (for complex by Veolia Water System) [19].

### 2.5. Treatment of Power Plant Waste Water, Hodonín Power Plant (250 MW, 105 MWe)

An ideal plant does not produce any waste. This is only a theory, which is obviously impossible to reach because it would involve perpetual motion. However, in the case of liquid waste, it is possible to get close to such a state by utilizing zero liquid discharge technology. This is the reason why ion exchange cannot be principally used for cooling tower blowdown recycling and waste-water volume reduction (as its regeneration produces further waste). The most suitable membrane process is electrodialysis again. 

**Figure 10:**
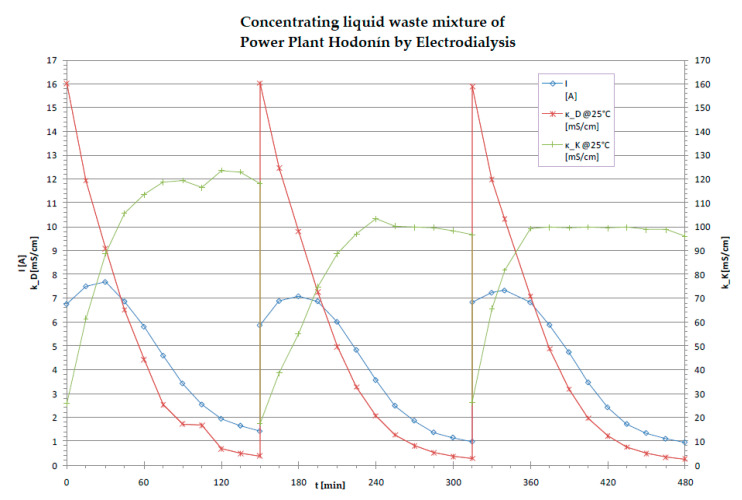
The concentrating of cooling tower blowdown (approx. 16 mS cm^−1^) mixed with all power plant waste-waters (final conductivity of dilutate kD 0.2 mS cm^−1^). This waste-water in the power plant Hodonín was treated in the pilot test by an EDR-Y electrodialysis unit (Mega co) in 3 cycles. The whole volume of the batches was 323 L, and the whole volume of final concentrate (made by electrodialysis) was 15 L (conductivity of concentrate k_K 100–120 ms cm^−1^).

**Figure 11:**
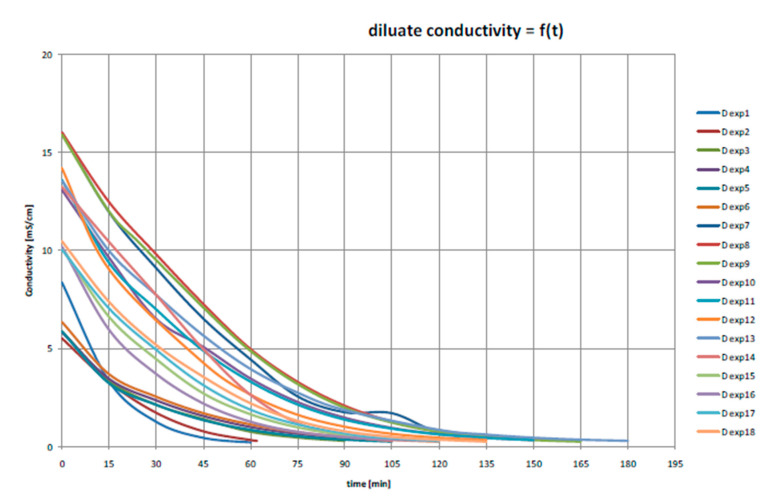
Time of achieving the required conductivity of diluate (0.3 mS cm^−1^) in 18 experiments.

The case of the waste-water treatment experiment (Figure 10; Figure 11 and Table 6) was carried out in the Hodoní Power Plant (ČEZ group) where the blowdown was mixed with all power plant waste-waters (from demineralization, flocculation, boilers blowdown, etc.). The inlet stream was treated only by decantation in waste water tank and then concentrated by electrodialysis to 2.5–4.6% of the original volume.

The diluate represents at least 90% (usually 95 to 97%) of inlet liquid waste volume, and it can be used as the make-up water for the demineralization plant (both ion exchangers and membrane processes). The quality of diluate is comparable with the salinity of the raw water (surface water), that is, 0.2 mS cm^−1^. The concentrate is usually around 3–5% of the inlet liquid water volume, and it can be used as an additional solution for the solidification (preparation of solid bricks in building construction industry). 

**Figure 12:**
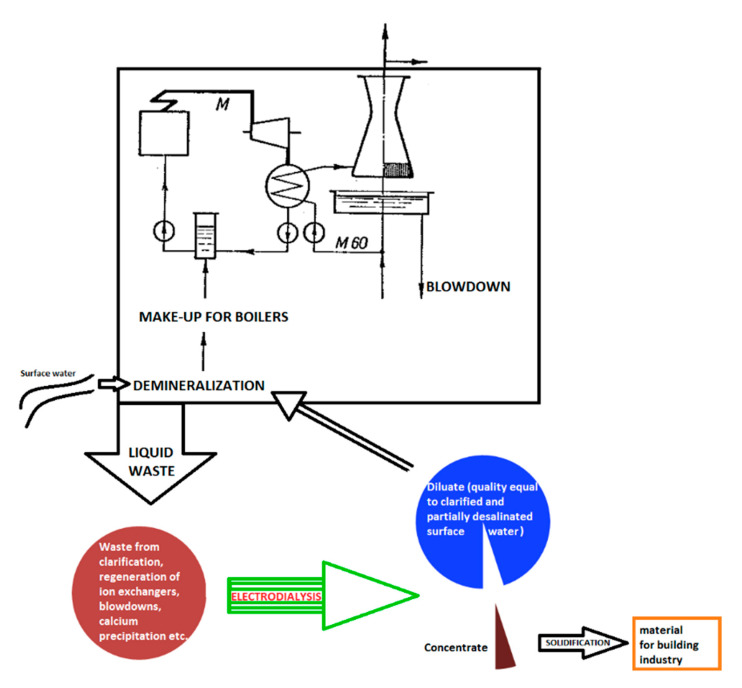
Treatment of mixture of liquid waste from power plant or heating station using combination of sludge filter press and electrodialysis: zero liquid discharge [1,6,8].

This case of liquid waste treatment was validated via a pilot test realized by MemBrain company in the Hodonín Power Plant, ČEZ Group. The pretreatment for the electrodialysis unit EDR-Y (by Mega co.) included decantation only. More than 300 liters of waste-water were treated in each experiment. In real operation, the decantation would be replaced by a chamber (or sludge) filter press (to reach zero liquid discharge, as described in Figure 12). In this pilot test, the operating costs for waste-water dropped from the original EUR 5.6 per 1 m^3^ (filtration, neutralization and other chemical processes, administration, fees for discharging waste-water, etc.) to EUR 0.2 per 1 m^3^ (approximated by electric power used for the electrodialysis stack—while the electric power is a marginal expense in the power plant). The waste-water reservoir volume was 100 m^3^.

### 2.6. Basic Overview of Eastern European Power and Heating Stations Using Membrane Technologies

The actual percentage of membrane technology utilized for water treatment in the power industry is shown below—for Czechia, Poland, Ukraine and Hungary.

Approximately 10 years ago, membrane processes started being used and tested for additional water treatment in Power plants in Czechia [6], as follows:

- waste-water treatment using RO in the Prunéřov Thermal Power Plant;
- colloidal removal using RO against the clogging of valves in the Temelín Nuclear Power Plant;
- utilization of RO to decrease the consumption of chemicals for ion exchange demineralization in the Ledvice supercritical unit;
- ED recycling of boric acid in the primary circuit of the Temelin Nuclear Power Plant;
- condensate demineralization using EDI in the Chomutov Heating Station;
- utilization of RO in three heating stations—Liberec, Chomutov and Žďas.

Furthermore, the situation in Poland is as follows:

- Lublin Wrotków Heating Station (with power production) using RO + EDI;
- Rzeszów Heating Station using RO + EDI;
- Chorzow using hybrid technology of ion exchange combined with RO.

In Czechia, there are 27 thermal power plants (>100 MW), two nuclear power plants and 73 heating stations, as follows:

- Two heating stations using RO (represents 3% of all Czech heating stations);
- Two power plants using hybrid systems (represents 7% of all Czech power plants).

As for Poland, there are 48 power plants and heating stations (coal, gas; >55 MW), as follows:

- Two heating stations using RO + EDI (representing 2% of all Polish heating stations);
- Two power plants using hybrid systems (represents 2% of all Polish power plants).

In Ukraine, there are 18 thermal power plants and four nuclear power plants. Five of them utilize membrane processes for the treatment of surface water from the Dnieper river. This constitutes 22%. There are also 32 heating stations, and membrane technology is utilized in water treatment in 15% of them [20].

In Hungary, there are 15 power plants and four nuclear power plants. Since 1992, membrane processes and hybrid membrane processes have been used (i.e., “RO + mixed bed” in the Sajószöged gas turbine plant and in the Litér gas turbine power plant). The combination of RO + EDI is more often used for make-up boiler water, while RO is employed in the treatment of cooling water for cooling towers. It also has a longer history (first installation in the 1990s). In summary, membrane processes are used in 48% of plants [21]. 

When the above numbers are summarized, they still constitute a negligible percentage (less than 20% for the whole of Eastern Europe) of the power and heating stations using and switching to membrane technologies.

## 3. Fouling of Membranes—Weakness of Membrane Processes

Higher water needs of inlet streams for membrane processes are not the only weak side of this technology (compared to IX). Another issue is fouling of membranes and ion concentration polarization (ICP). Fouling of membranes for pressure processes (such as RO, NF, UF, MF membranes) is not discussed in detail because their fouling and ICP can be strongly affected by controlling the crossflow (i.e., velocity of the solution flowing along the membrane). If the crossflow is high enough, the lifetime of membranes far exceeds the guaranteed operating life (4 years). There are cases of reverse osmosis membranes exhibiting more than 15 years of smooth operation [17]. Several laboratories work on developing special antifouling materials, e.g., polyethersulfone hollow-fiber ultrafiltration membranes doped with nanosilver [22]. However, in real-life water treatment with quality engineering and the adjustment of the right crossflow, this research direction does not make sense and shows a misunderstanding of the whole process of ultrafiltration.

In the case of electromembrane processes, the fouling is a little bit different—ion exchange material showed to be a good substrate (cultivating medium) for bacteria and fungi (especially when treating beverages or whey) [23]. There are a few works regarding research into the antimicrobial properties of quaternary ammonium groups (i.e., anion exchange membranes) [24], and membranes doped with silver [23,25]. The comparison of bacterial growth on the surface of anion vs. cation exchange membranes is shown in Ref. [26], with a surprising result that cation exchange membranes have better antifouling properties compared to anion exchange membranes. A detailed study on the fouling of anion exchange membranes was made by the Wetsus company [27].

## 4. Optional Novel Methods

Four main representative processes were selected as optional future methods of desalination. The first of them, capacitive deionization (CDI), could potentially replace EDI. The RO + CDI combination thus constitutes an optional system for ultrapure make-up water for boilers. The specific energy consumption of EDI commonly ranges from 0.39 to 2.11 kW m^−3^, while for CDI, it is 0.02–0.22 kW m^−3^. However, for each particular case the EDI consumption is lower due to the utilization of membranes and resin in between the electrodes, which together decrease the resistance of deeply deionized water [28]. Moreover, the so-far maximum desalination reached by the CDI electrochemical method, which operates below the potential of water electrolysis to avoid byproducts (theoretically 1.2 V; approx. 2.4 V for surface water or 1.4 V for permeate from pressure membrane processes), was 78–92% desalination efficiency, while EDI can provide 99.9999% desalination efficiency. This makes it a suitable process for the softening of water or heavy metal removal [13,29]. The recent trends bring about the development of electrodes in flow form [30].

The second process is forward osmosis (FO). Compared to RO, FO is successfully applied for waters with 70.000 ppm and higher salinity, and for such waters the energy consumption is 29.45–29.91 kW m^−3^ [13,31]. This process might therefore be used for the pretreatment of salty water (sea water) before reverse osmosis, but it still utilizes the same membranes as RO, so FO needs a pretreatment by means of UF or MF. The energy consumption can be reduced by installing the technology under the water level, thus utilizing the hydrostatic pressure as a power input, but the piping, maintenance and pretreatment are very complicated. It is a suitable process for wastewater treatment in the oil and gas industry, the separation of boric acid from the primary circuit in nuclear power plants [32] or wastewater treatment in the mining (heavy metals) industry, but is irrelevant for wastewater treatment in the power industry, as it results in high water discharge [30,31].

**Figure 13:**
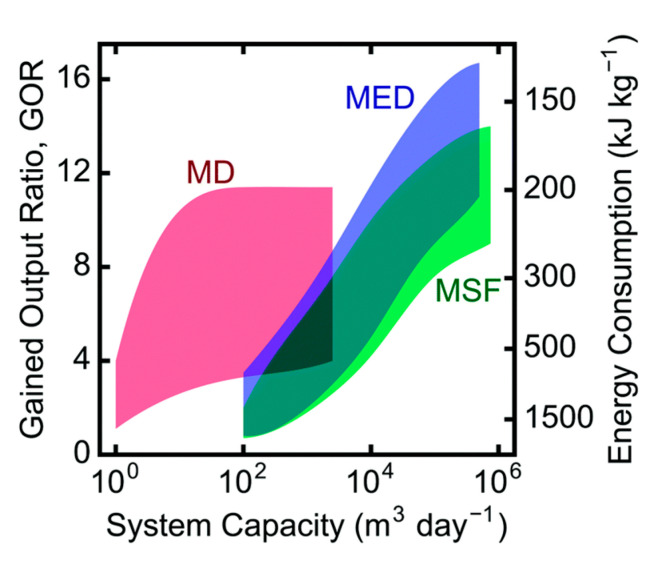
Distillation energy requirements (MD—membrane distillation, MED—multiple-effect distillation, MSF—multistage flash distillation) [34].

The third representative of an optional process for water treatment in the power industry is membrane distillation (MD). The number of articles concerning this process is growing exponentially. In fact, membrane distillation is still just a distillation process (even in the form of MSF (multistage flash distillation) or MEE (multiple-effect evaporation) vapor compression (VC) [9,30]), which is the most power-demanding water treatment process (ranging from 2.03 to 47.41 kWh m^−3^ of electricity consumption, plus 45.38 kWh m^−3^ of thermal consumption; see Figure 13). Power plants, however, abound in surplus power. This can be used with advantages for high-salinity solutions. The rejection of non-volatile compounds is 100%. However, as of today (2020), the cost of modules is pretty high and the flux is very low. Moreover, the technology also requires a larger built-up area. The process is not affected by fouling. It is thus suitable for beverage production or the removal of volatile components, paradoxically (e.g., ammonia) [33].

Despite being able to theoretically reject all non-volatile solutes (i.e., salts), the main drawback of the MD process is the large amount of energy that is consumed during the liquid–vapor phase change process, which, coupled with the incomplete recovery of the latent heat, renders the MD process energy-inefficient as a standalone system. Nevertheless, MD’s ability to leverage low-grade waste heat as an energy source while operating at a low pressure, and its negligible sensitivity to varying feed salinity, merit consideration over conventional pressure-driven membrane processes for its application in water recovery from high-salinity feed streams, such as brines from produced water. The membranes have already passed through a long process of improving special surface wettability, and the next important challenge for future optimization is maximizing the porosity and optimizing the thickness of the membrane to minimize energy consumption, regardless of the configuration of the process (direct MD, Air Gap MD, Vacuum MD or Sweep Gas MD) [34,35].

**Figure 14:**
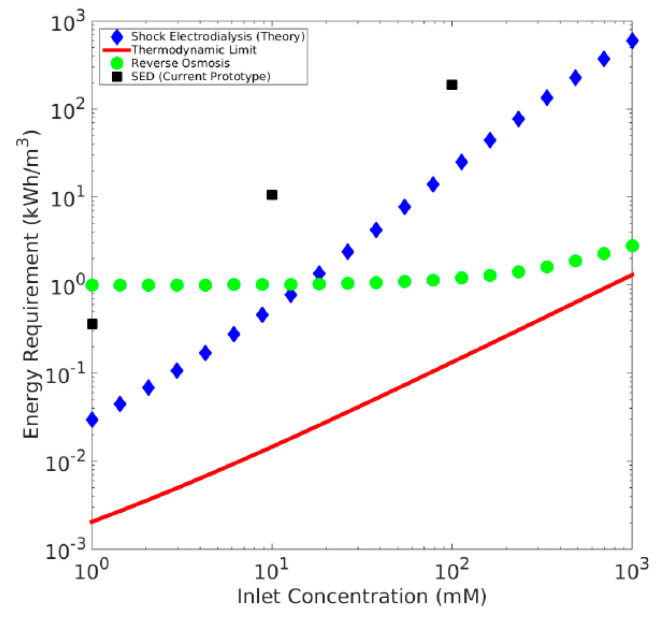
Very first calculation of energy requirements for SED, calculated and measured by Schlumpberger (Bazant‘s group at M.I.T., 2015) and compared to other desalination technologies achieving 99% ion removal and optimal water recovery [43]. The group have better energy efficiency in the recent papers of Alkhadra for desalination and selective separations [42], although not plotted in the same way vs RO.

The last, but not the least important, issue to consider is a shock electrodialysis process whose theoretical foundations were laid by the group of professor Nikonenko, (Kuban State University, Russia) [36], and the process was experimentally executed by Bazant’s group at M.I.T., (Cambridge, USA) [37,38] and later by Marek’s group at Technical University of Liberec, (Czechia) [39,40,41]. The theory says that the diluate stream is collected from the enhanced boundary layer of the membrane, which exhibits a very low concentration of rejected ions. It should thus be possible to desalinate the solution of ions regardless of the concentration, and obtain ultrapure water in a single step. However, as of today (2020), there are only laboratory prototypes (producing tens of ml h^−1^ at maximum) [39,42]. The Fujifilm company is developing the first bigger prototypes, but the results are still not easily reproducible and the process requires pretreatment by means of UF or MF (because of utilizing porous material between membranes, which enables so-called shock waves in its pores.) As there is additional material between the electrodes, the consumption of electricity is naturally higher (see Table 7 and Figure 14) compared to capacitive deionization (depending on the type of the selected porous material). As it is needed to reach a shock wave, cross the over-limiting current and achieve water splitting, it still requires fairly high voltage (10 to 30 V per chamber, dimensionless current of up to 5). The energy consumption (of TUL unit with non-optimized charge of porous media) for desalting 14 mS cm^−1^ to 2 mS cm^−1^ has been 900 Wh dm^−3^ so far, while for standard electrodialysis (for the same conditions, using Na_2_SO_4_ solution at the room temperature of 24 °C) the consumption is 4.6–4.9 Wh dm^−3^ (Table 7). Desalination efficiency of small prototype by MIT Bazant‘s group for artificial seawater was 99.8% [42] and (99.87 ± 0.09)% by TUL Marek’s group for Na_2_SO_4_ [41]. Regarding energy consumption compared to ED and regarding desalination efficiency compared to EDI (99.9999%) [40] the process still requires further optimization before scale-up for industrial utilization.

Another potential advantage of shock electrodialysis lies in its utilizing only one type of membrane (e.g., a cation or anion exchange membrane). As the preparation of anion exchange membranes is more complicated, and they are more sensitive to physical (thermal) and biological degradation (see Section 2.4), the cation exchange membranes would be sufficient for this process. This could simplify the production process.

## 5. Optional Novel Materials

As stated in the previous paragraph, there is only one combination of membrane processes useful for ultrapure water preparation which makes economic sense at present. It is UF (MF) + RO (NF) (double-pass or more) + EDI. The proof of this statement lies in the number of installations utilizing this technology in such a combination over the world, with capacities ranging from single units of cubic meters per hour to thousands per hour (e.g., 1.530 m^3^ h^−1^ in New York Combined Power Plant) [30,44].

The contracted desalination capacity of membrane processes increased from 1 million m^3^ d^−1^ in 1990 to its maximum of 7.25 million m^3^ d^−1^ in 2007, and since then it has been oscillating between 2 and 5 million m^3^ d^−1^. CapEx investments in membrane desalination processes worth USD 41.3 billion worldwide are planned in the upcoming years (2020–2024). The majority of these investments is supposed take place in the Near East (Saudi Arabia USD 10.8 bn, UAE USD 6 bn, Kuwait USD 3 bn, Oman USD 1.8 bn, Qatar USD 1.2 bn, Bahrain USD 0.1 bn and the rest of the world USD 18.4 bn) [45]. This means that the Near East is switching to membrane technology, while nowadays (2020), major desalination technologies are represented by RO (69%), MSF (multi-stage flash) (18%; 44.4% in 2010), MED (i.e., multi-effect distillation) and other thermal methods (7%; 8.4% in 2010), NF (3%), ED (2%), and others (1%) [46,47].

It is thus clearly visible that UF, MF, RO, NF, ED and EDI represent the major membrane technologies in water treatment, proved by long time in industrial operation and manufactured by number companies worldwide. UF and MF are not included in the calculation because they do not desalinate, but are essential as a pretreatment for RO, and finally, EDI is not included, because it represents final ultrapure water polishing (after RO). The rest of the technologies—FO, MD, CDI and others—constitute less than 1%. This means that the current trends in company research (not academic research) lead to the optimization of components for these major technologies.

**Figure 15:**
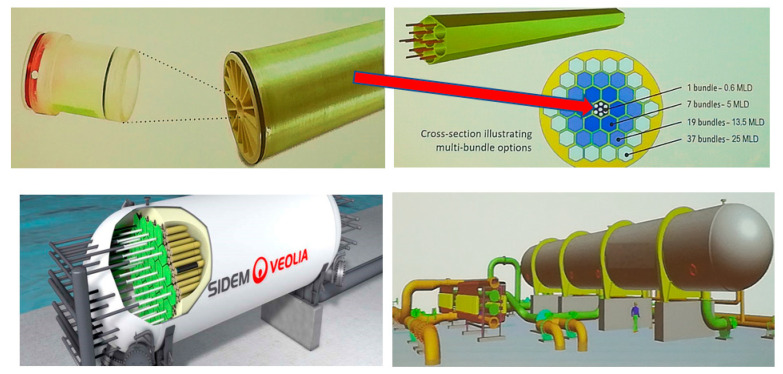
A huge “Barrel installation of RO plant” (lower right pic—showing comparison with a human body) with smart sensor (left upper pic) able to disable a defective module. Designed by Veolia [45].

These trends bring about RO membranes containing a wide variety of nanoparticles, including zeolites (silicate and NaA, NaX, NaY—i.e., different silica–alumina ratio), carbon materials (including carbon nanotubes and graphene oxide), silica, metal oxides (titanium dioxide), metal nanoparticles, and there has even been an attempt to introduce novel organic–inorganic hybrid materials (ZIF-8) into aromatic polyamide layers of RO membranes [48]. Special newly developed thin film composite biomimetic membranes greatly increase the effectivity of water transport in RO membranes, and increase desalination effectivity [49]. Additionally, according to the huge desalting RO plants, huge “kits” or “sets of blocks” of modules are being developed (Figure 15).

As regards ED, for example, the pulsed electric field technology increase the desalination velocity (leading to higher demineralization) very significantly (10–20%), and offers perfect antifouling properties [50]. And also membrane spacers are still in optimization process [51].

In the last 100 years, there have also been many trials, and the development of materials of both heterogeneous and homogenous ion exchange membranes. Heterogeneous membranes are more mechanically durable, while they have worse transporting (kinetic) properties. However, their mechanical stability is used with advantages in suitable technologies. The mechanical stability can even be enhanced by crosslinking the inert binder [52]. The preparation of microfibrous ion exchange membranes (Mion from Promion company in Kaluga, Russia, which does not exist anymore), Fiban (National Academy in Belarus), or Johnsson Matthey (originated in Norway) has showed itself as the most effective means of modification of homogenous membrane form. The next level of fibrous membranes were the ion exchange nanofibers developed in professor Chase’s group, and included within the author’s dissertation thesis. Although the kinetic properties of the membranes are exceedingly good, they have never found their place in actual industrial utilization because of their rather high preparation costs and poor mechanical properties [53,54].

EDI efficiently uses bipolar membranes, increasing water splitting, which enhances the desalination effectivity [55,56]. It is possible to utilize ion exchange resin in filling in the form of a layered bed, a separated bed or a mixed bed. One could even use only cation exchange resin or anion exchange resin [53]. The importance of pretreatment with double-pass RO for long-term operation has already been proven (mentioned above). The benefits of spiral-wound EDI were summarized by Dey and Tate [57].

This means that the components in each process, including RO, NF, ED and EDI, can be further optimized, each component can be easily replaced (RO starting on acetate cellulose membranes followed by polyamide and currently biomimetic membranes), and EDI can use just cation resin as the packed bed in between the membranes (the sealing, piping, stack fixing, supporting textiles, electrodes, pumps, valves, cartridge prefilters, heterogeneous to homogenous membrane (i.e., Ralex or FumaTech heterogeneous to Nafion homogenous membranes or penta-block copolymers from Kraton), etc.) [58].

If SED is successfully optimized and employed in producing ultrapure water, it can use just cation exchange membranes, but more likely it will use just anion exchange membranes due to their higher effectivity in water-splitting [41].

## 6. Conclusion of Current and Novel Technological Models for Water Treatment in Power Sector

To make survey information of the industry-proven water treatment methods in power sector, detaily described in Section 2, all the outputs were summarized in Table 8. 

The outputs of Section 3, Section 4 and Section 5 which presents optional novel technologies, mostly without final technical and economic reasonability for industry utilization, just only according to their theoretical prediction, are concluded in Table 9.

## 7. Conclusions

The power industry represents the biggest share of the consumption of water resources in most European countries. If we want to save water sources, minimize waste-water discharge and change the consumption of water, the most effective way would be to focus especially on this industry. This work shows complex summary of the common and novel environmentally friendly, economic and reasonable water treatment methods in power sector (not only) for Czechia, preserving environment, saving money and bringing new technical benefits. 

It is well-known that the membrane processes are the state-of-the-art for the most economical and ecological water treatments (not only) in the power generation sector. The membrane processes are represented by pressure membrane processes and electromembrane processes. Both processes are essential for power generation.

The portfolio of membrane processes is growing each day. Although the majority of novel methods, including membrane distillation, capacitive deionization or forward osmosis, do not meet the technical and economical requirements yet, and need further investigation, it is still possible to choose from well-established membrane technologies such as reverse osmosis, nanofiltration, ultrafiltration, microfiltration, electrodialysis and electrodeionization.

Membranes are more economical, environmentally friendly, and simple to operate, bringing many technical benefits and taking up less built-up space compared to ion exchange technology. Though they have some weak spots, such as fouling, these can be avoided by the correct design of the particular process (increased crossflow, etc.) or by the further investigation of antifouling materials. The ion exchange technology is quite old, and is neither ecological nor economical. Despite that, when designing new power plants or extending the current operation, the ion exchange technology is still the primary solution in Czechia and other Eastern European countries. This work suggests that the major aspect should be the environmental impact. Obviously, each company decides on which technology to use based on the economy of investment and operation. However, even this criterion already speaks for membrane technologies.

Western Europe has already begun transforming water treatment technologies in power generation to membrane processes. This is the time for the rest of Europe to follow, and to change the current technologies, not even to save money but primarily to protect nature. There are no more arguments for stagnation by ion exchange technology. On the contrary, there are many economic, ecological and technological arguments for the substitution of ion exchange technologies.

The novelty of this article lies in the completion of realistically usable innovative-type technologies for all circuits in the power plant. Each water cycle in power generation has its own ideal treatment technology, as shown in Table 8 and Table 9. This article showed case studies and real operation technologies proving all these statements. 

Worldwide used ideal combination of membrane water treatment technologies for boiler make-up water is UF + 2(RO) + EDI. The waste water is efficiently recycled by ED (including blowdowns and other liquid waste mixtures), the same process (ED) is used for concentrating boric acid in primary circuits. Additionally, the study of TOC rejection revealed that the combination of RO + EDI is more appropriate for the prevention of TOC contaminants in make-up water compared to RO + mixed bed. There are examined novel technological models, such as the efficient treatment of turbine condensate using sand filter + EDI and waste-water treatment by means of a sludge filter press and ED, which has not been shown anywhere else yet. The last combination reduces waste-water to up to 3–5% of its original volume.

Novel methods promise valuable benefits such as 100% desalination of water by MD, efficient concentrating of boric acid by FO or utilizing electrolysis without necessity to manufacture the membranes (CDI). There is even a vision of utilizing only one type of single charge membranes (in SED) or latent heat in power plants (by MD). While two last mentioned processes should theoretically produce ultrapure water, the experimental results still have not reached the theoretical presumptions. This is why EDI is currently unavoidable process for the continual and reliably safe production of ultrapure water in power generation. The proof of this statement is that (to date) there is no power plant globally using SED, CDI, FO or MD for the production of ultrapure water.

The novel methods and materials, including CDI, FO, MD and SED, still have a long way to go in order to be utilizable in the power industry. Although they are not completely novel, as their foundations were laid more than 50 years ago, they still need further investigation and optimization for real and safe utilization.

## Figures and Tables

**Table 1:** Millions of cubic meters of water consumption in Europe * per year [2].

Millions of m^3^ y^−1^	Energy	Industry	Agriculture	Public Water Supply
Eastern				
Early 1990s	21,901	12,573	13,945	11,058
Latest year	18,538	4882	3545	5990
Percent.	56%	15%	11%	18%
Western				
Early 1990s	67,088	22,548	7570	33,682
Latest year	54,787	17,787	5797	29,439
Percent.	51%	16%	5%	27%
Southern				
Early 1990s	6635	2010	35,542	13,828
Latest year	7018	666	33,175	16,738
Percent.	12%	1%	58%	29%
Turkey				
Early 1990s	67	734	17,842	3235
Latest year	98	810	40,643	5792
Percent.	0%	2%	86%	12%
Europe	80,441	24,145	83,160	57,959
	33%	10%	34%	24%

*: Water abstractions data are not available for all sectors and periods. Eastern Europe: Bulgaria, Czechia, Estonia, Latvia, Lithuania*, Hungary, Poland, Romania, Slovenia, Slovakia. Western: Belgium, Denmark, Germany, Ireland*, France, Luxembourg, Netherlands, Austria, Finland, Sweden, England and Wales, Iceland, Norway, Switzerland*. Southern: Greece, Spain, Italy*, Cyprus*, Malta, Portugal*. Turkey is plotted on an individual column in this graph to depict the large increase in water use.

**Table 2:** (**a**,**b**) Consumption of chemicals by IX (ion exhchange) technology in the Liberec Heating Station (first half of 2010). RW–raw water, DEMI–outlet of IX.

(**a**)					
**Season**	**Raw Water**	**HCl**	**NaOH**	**Consumption of Chemicals**	
Jan–Jun	41 m^3^ h^−1^	366 g m^−3^	293 g m^−3^	660 g m^−3^ per RW	418 g m^−3^ per DEMI
(**b**)					
**Price 31% HCl + 50% NaOH**					
0.08 EUR m^−3^ per RW			0.05 EUR m^−3^ per DEMI		

**Table 3:** CapEx of IX vs. RO + EDI (2010) (EDI of company Mega constitutes former EDI-X modules, which have been recently substitute by MPure^TM^ technology).

EUR	IX (Parallel Flow Regeneration)	IX (Counterflow Regeneration)	RO + EDI Culligan	RO + EDI Mega
Mechanic supply	116,259	89,333	37,815 + 55,185	70,296 + 74,444
M&R	24,407	8926	9296	13,852
Mechanic and M&R assembly	20,741	14,741	9296	15,630
Mechanic and M&R project	8185	5222	4667	17,407
Startup	7037	4630	4667	3963
Total	176,593	122,889	120,889	195,630

**Table 4:** OpEx of IX vs. RO + EDI (2010).

Production of Demiwater (m^3^ y^−1^)	3000	4500	6000	7500	9000	10,500
**1. IX counterflow regeneration, higher controlling system**						
chemicals (EUR)	1420	2130	2840	3550	4259	4969
water expensis (EUR)	6290	9434	12,579	15,724	18,869	22,013
total (EUR)	7709	11,564	15,419	19,273	23,128	26,983
**2. IX parallel flow regeneration, autonomic cooperating systems**						
chemicals (EUR)	3060	4590	6120	7650	9180	10,710
water expensis (EUR)	6290	9434	12,579	15,724	18,869	22,013
total (EUR)	9349	14,024	18,699	23,374	28,048	32,723
**3. RO + EDI**						
chemicals (EUR)	33	50	67	83	100	117
water expensis (EUR)	6889	10,333	13,777	17,221	20,666	24,110
total (EUR)	400	600	800	1000	1200	1400

**Table 5:** Exact entries of membrane and IX technologies from Figure 6.

EUR m^−3^		IX	Membrane Technology		
			UF	RO	EDI *
Energy	pumps	0.0122	0.0107	0.0326	0.0093
	voltage	0	0	0	0.0137
Chemicals	HCl, NaOH	0.0837	0	0	0
	Antiscalant *	0	0	0	0
	NaClO	0	0.0019	0	0
Safety filters	bougie (5 μm)	0	0	0.0002	0
Water expenses		0.1344	0.1344	0.1344	0.1344
Subtotal **		**0.2304**	0.1470	0.1673	0.1574
RO + EDI				**0.1903**	
Exchange of membrane modul		0	0.0011	0.0041	0.0341
Exchange of resin		0.0326	0	0	0.0011
Work ***, maintanance, CIP		0.0156		0.0085	
OpEx total	**IX**	**0.2785**	0.1481	0.1800	0.1926
	**RO + EDI**			**0.2369**	
	**UF + RO + EDI**			0.2507	
Depreciation		0.0985	0.0470	0.0704	0.0333
Total expenses for	**IX**	**0.3770**	0.1952	0.2504	0.2259
water production	**RO + EDI**			**0.3396**	
	**UF + RO + EDI**			0.4011	

** The subtotal value is always lowered by water expenses (because of one water stream), *** work is calculated from average salary of heating station employees (550 EUR m^−1^), * antiscalant was not needed (because of LSI of RW river Nisa) and * EDI technology composed of EDI-X modules (former technology of Mega company, which has been recently upgraded to MPure^TM^ technology).

**Table 6:** Consumption of electric power for each experiment. The inlet waste-water started at 5.5 mS cm^−1^ (2nd to 6th experiment, carried out in a lab) and reached its maximum at 16 mS cm^−1^ (for 8th experiment).

Experiment (No.)	t (min)	E (Wh/kg RAW Salt)	Inlet (mS/cm)
**01**	60	2.91	8.4
**07**	150	6.07	16.0
**08**	165	5.44	16.0
**09**	165	5.75	15.9
**10**	150	4.22	13.1
**11**	60	5.16	13.6
**12**	135	3.56	14.2
**13**	180	4.86	13.6
**14**	105	4.85	13.2
**15**	120	3.57	10.2
**16**	120	3.88	10.2
**17**	120	3.92	10.0
**18**	135	6.24	10.5

**Table 7:** Comparison of operating parameters of standard vs. shock electrodialysis reversal (standard lab unit EDR-Z/10 by Mega co. and pilot shock ED unit by Technical University of Liberec, generation IV module—without optimization of charge of porous media) [40].

Parameter		Unit	Module	
			EDR-Z/10 (Mega Co.)	Pilot SED (TUL, Non-Optimized Porous Media)
Number of membrane pairs		(-)	10	1
Membrane active surface		(cm^2^)	64	50
Total active surface of membrane		(cm^2^)	640	50
Thickness of working chamber		(mm)	0.8	10
Voltage per membrane pair/working chamber		(V)	1	≈30
Specific production of diluate	14.5 mS cm^−1^ -> 2 mS cm^−1^	(dm^3^ dm^−2^ h^−1^)	0.19–0.41	0.024
	14.5 mS cm^−1^ -> 6 mS cm^−1^		0.45–0.76	0.12
Specific elektricity consumption	14.5 mS cm^−1^ -> 2 mS cm^−1^	(Wh dm^−3^)	4.6–4.9	900
	14.5 mS cm^−1^ -> 6 mS cm^−1^		3.2–3.6	500

**Table 8:** Industry proven water treatment processes in power generation sector regarding their technical and economic feasibility (list of abbreviations bellow, page 25).

Industry-Proven Methods with Reasonable Economics								
Makeup water for boilers	According to pressure	Inlet water quality	SDI_15_	Outlet water quality	UF (MF)	RO (NF)	EDI	ED
	Low & Mid (<8 MPa)	municipal water, well water, after quality clarification (coagulation)	<5	according to ČSN and EN				
		surface water, low quality clarification	>5		+			
		municipal water, well water, good clarification	<5	better than ČSN and EN		+		
		surface water, low quality clarification	>5		+ +			
	High (≥8 Mpa)	municipal water, well water, good clarification	<5	according to ČSN and EN		+		
		surface water, low quality clarification	>5		+ +			
	Turbine	municipal water, well water, good clarification	<5			+		
			>5					
Recyclation of cooling water		municipal water, well water, good clarification	<5	control of LSI, RIS!!!				
		surface water, low quality clarification	>5					
Waste waters		liquid waste mixture	COD < 100					
Recyclation of turbine condensate		after send filter		According to ČSN and EN				
Recyclation of boric acid in primary circuit								
Rejection of colloidal substances in secondary circuit								

**Table 9:** Potential novel methods of the water treatment in power generation sector, regardless their economic and technical characteristics, just only according to their theoretical presumptions (list of abbreviations bellow, page 25).

Industry-Proven Methods With Reasonable Economics					Potential Novel Methods (Regardless Economy)			
Makeup water for boilers	According to pressure	Inlet water quality	SDI_15_	Outlet water quality	CDI	FO	MD	SED
	Low & Mid (<8 MPa)	municipal water, well water, after quality clarification (coagulation)	<5	according to ČSN and EN				
		surface water, low quality clarification	>5		UF + CDI	UF + FO	UF + MD	UF + SED
		municipal water, well water, good clarification	<5	better than ČSN and EN	RO + CDI	FO + EDI		
		surface water, low quality clarification	>5		UF + RO + CDI	UF + FO + EDI	UF + MD	UF + SED
	High (≥8 Mpa)	municipal water, well water, good clarification	<5	according to ČSN and EN		FO + EDI		
		surface water, low quality clarification	>5			UF + FO + EDI	UF + MD	
	Turbine	municipal water, well water, good clarification	<5			FO + EDI		
			>5			UF + FO + EDI	UF + MD	
Recyclation of cooling water		municipal water, well water, good clarification	<5	control of LSI, RIS!!!				
		surface water, low quality clarification	>5					
Waste waters		liquid waste mixture	COD < 100					
Recyclation of turbine condensate		after send filter		According to ČSN and EN				
Recyclation of boric acid in primary circuit								
Rejection of colloidal substances in secondary circuit								

## Data Availability

MDPI Research Data Policies.

## Abbreviations

ρ	Specific resistance = electric resistivity (MΩ cm^−1^ at 25 °C)
κ	Specific conductance = electric conductivity (μS cm^−1^ at 25 °C)
CDI	Capacitive deionization
CIP	Clean in Place
COD	Chemical oxygen demand
ED	Electrodialysis
EDI	Electrodeionization
FO	Forward osmosis
ICP	Ion concentration polarization
IX	Ion exchange technology
LSI	Langelier saturation index
MD	Membrane distillation
MED (MEE)	Multi-effect distillation (evaporation)
MF	Microfiltration
M&R	Measurement and regulation
MSF	Multi-stage flash
NF	Nanofiltration
RO	Reverse osmosis
RIS	Ryznar index of stability
RW	Raw water (i.e., surface water—river or lake, κ up to 1.5 mS cm^−1^)
SDI_15_	Silt density index (time period 15 min, 450 nm cellulose filter, 1 bar)
SED	Shock electrodialysis
TOC	Total oganic carbon
UF	Ultrafiltration
VC	Vapor compression
